# Can we achieve pain stratification in musculoskeletal conditions? Implications for clinical practice

**DOI:** 10.3389/fpain.2024.1362757

**Published:** 2024-03-07

**Authors:** Nidhi Sofat, Andrew Lambarth

**Affiliations:** ^1^ Institute for Infection and Immunity, St George’s, University of London, London, United Kingdom; ^2^ Department of Rheumatology, St George’s University Hospitals NHS Foundation Trust, London, United Kingdom

**Keywords:** rheumatic and musculoskeletal diseases, nociceptive pain, neuropathic pain, nociplastic pain, pain sensitisation

## Abstract

In the last few years there has been an increased appreciation that pain perception in rheumatic and musculoskeletal diseases (RMDs) has several mechanisms which include nociceptive, inflammatory, nociplastic and neuropathic components. Studies in specific patient groups have also demonstrated that the pain experienced by people with specific diagnoses can present with distinctive components over time. For example, the pain observed in rheumatoid arthritis has been widely accepted to be caused by the activation of nociceptors, potentiated by the release of inflammatory mediators, including prostaglandins, leukotrienes and cytokine networks in the joint environment. However, people with RA may also experience nociplastic and neuropathic pain components, particularly when treatments with disease modifying anti-rheumatic drugs (DMARDs) have been implemented and are insufficient to control pain symptoms. In other RMDs, the concept of pain sensitisation or nociplastic pain in driving ongoing pain symptoms e.g. osteoarthritis and fibromyalgia, is becoming increasingly recognised. In this review, we explore the hypothesis that pain has distinct modalities based on clinical, pathophysiological, imaging and genetic factors. The concept of pain stratification in RMD is explored and implications for future management are also discussed.

## Introduction

**Figure 1:**
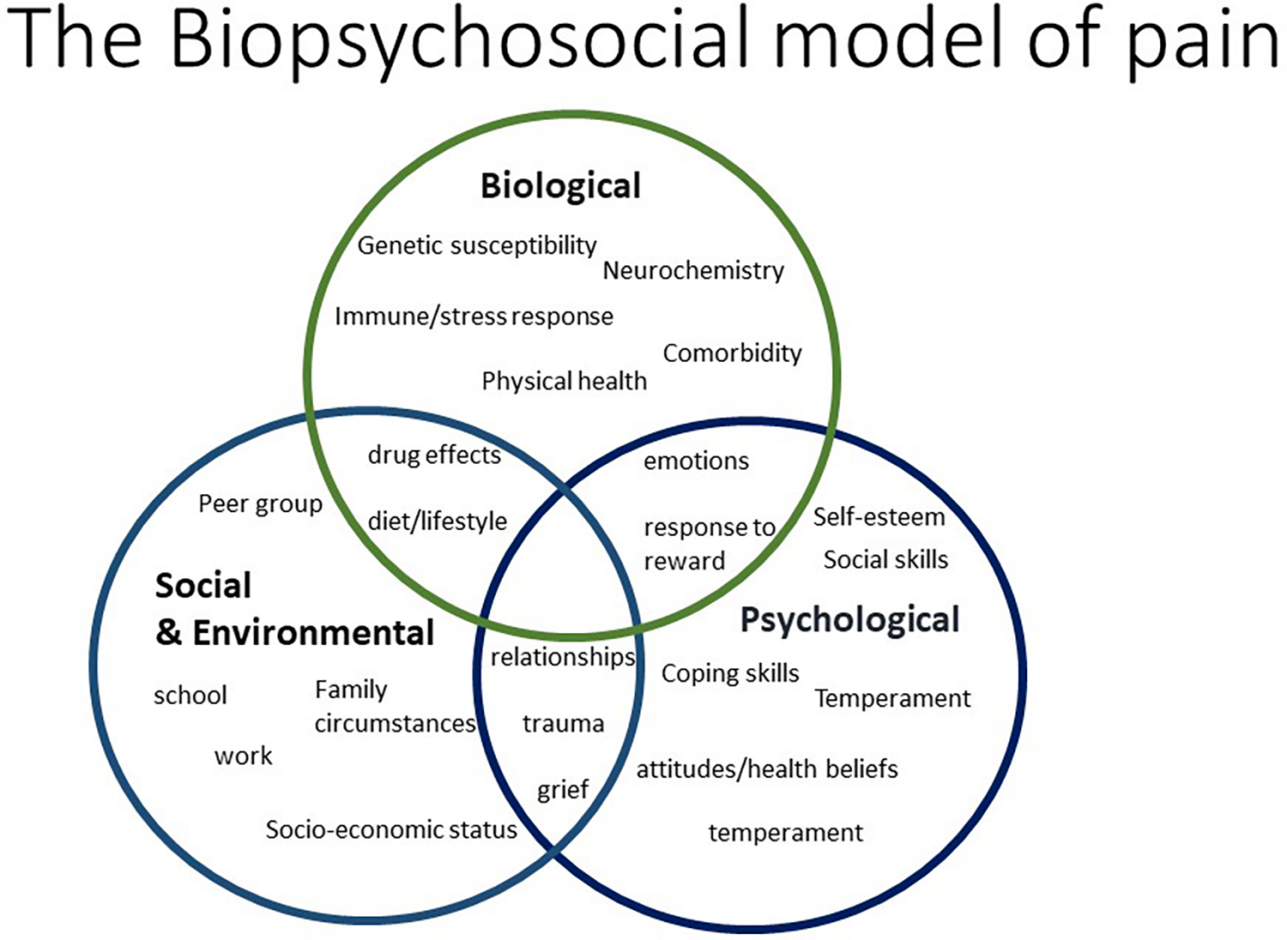
Schematic representation of the biopsychosocial model of pain.

Chronic pain is a major health burden in the UK, estimated to affect almost one third to half the population at any time (1). Fayaz et al. (1) reported that up to 28 million UK adults are affected by chronic pain, with the most common conditions contributing to chronic pain including chronic widespread pain (CWP) (14.2%), chronic neuropathic pain (8.2%–8.9%) and fibromyalgia (5.4%). Factors influencing the development of chronic pain include age, sex, occupation, ethnicity, social background and psychological factors (2). The complex pathophysiology influencing chronic pain development is often termed as the biopsychosocial model of pain (3–5) (Figure 1). Some of the most common pain disorders include rheumatic and musculoskeletal diseases (RMD). In this review we will use RMD as an exemplar for discussing pain mechanisms. By gaining a deeper understanding of pain mechanisms, we might be able to improve diagnosis and treatments for people with chronic pain.

### How do people with rheumatic and musculoskeletal diseases (RMD) experience pain?

**Figure 2:**
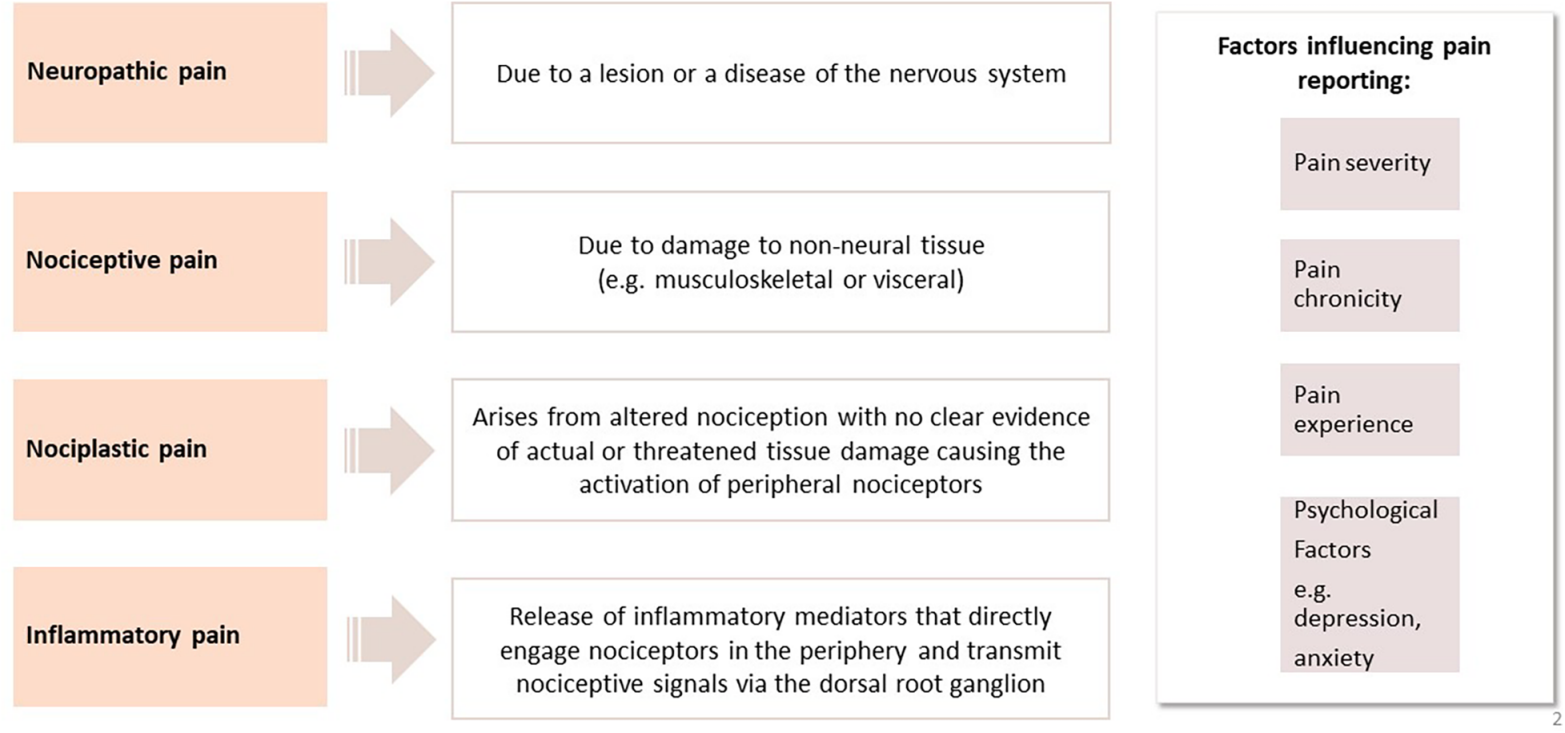
A summary of pain mechanisms in rheumatic and musculoskeletal diseases and factors influencing pain.

When considering the burden of conditions associated with chronic pain, RMD feature highly. The most common RMD contributing to the statistics of chronic pain include back pain, osteoarthritis (OA), fibromyalgia and autoimmune inflammatory disorders e.g., rheumatoid arthritis and psoriatic arthritis (6). Although the conditions described are considered to be their own entities with specific diagnostic criteria in many cases, symptoms of pain rate highly in all. Such observations have led to the consideration of what the underlying mechanisms of pain are in these conditions and have led researchers to consider if there may be distinct and also common shared mechanisms of pain in RMD. By gaining a deeper understanding of pain mechanisms in RMD, this would help to identify features of pain which may then be amenable to targeted therapies. We will focus on specific characteristics of pain, including inflammatory, nociceptive, nociplastic and neuropathic pain and explore how such descriptors are recognised in specific RMDs (Figure 2).

### Nociceptive pain

As a human species, we are designed to experience pain in response to noxious stimuli e.g., heat, cold, intense mechanical stimuli or chemical irritants (7). Nociceptive pain is an early warning system that has been maintained throughout evolution to protect us from noxious stimuli and as such is essential for maintaining the body's integrity. Our ability to respond to noxious painful stimuli is described as nociceptive pain. Sometimes analgesics may be used to reduce nociceptive pain e.g., post-surgery and can be very effective for short periods (8). However, it is important to be careful about not inhibiting nociceptive pain to such an extent that the protective role of nociceptive pain is lost (9).

### Inflammatory pain

Acute pain is often characterised by the release of inflammatory medicators, which leads to clinical features of redness, swelling, heat and ultimately pain (10). The underlying pathological processes driving acute inflammation include the release of inflammatory mediators, including cytokines, prostaglandins, leukotrienes and chemokines, coupled with an influx of inflammatory cells, including neutrophils, macrophages and mast cells (11). Release of inflammatory mediators can directly engage nociceptors to activate pain networks in the periphery e.g., a joint, with transmission of nociceptive stimulation to the neuronal cell bodies through activation of the dorsal root ganglion.

Cells and their released proteins work rapidly to resolve inflammation and the activation of nociceptors with it. Recent work has suggested that chronic pain may continue when inflammation e.g., mediated by neutrophils does not resolve rapidly, and may even persist after inflammation apparently resolves (12). In other people with no inflammatory stimulus, chronic pain may develop *de novo* and is described as pain that persists for more than 3 months (13). Many people with RMD may have persistent stimulation of inflammatory pathways that leads to ongoing activation of pain networks (14, 15). Genetic predisposition for the transition of acute pain to chronic pain e.g., back pain, has also become increasingly recognised as an important risk factor, and mechanistically may have inflammatory underpinnings (16).

### Neuropathic pain

In neuropathic pain, there is chronic activation of pain networks which results from abnormal functioning of the nervous system. This form of pain is considered maladaptive since it is not protective and can lead to significant burden of pain symptoms. Examples of neuropathic pain include conditions such as spinal cord injury, which lead to chronic pain activation (17). Low back pain and associated radiation into the legs, often termed sciatica, causing radicular pain is a neuropathic leg pain secondary to compressive lumbosacral nerve root pathology, which may be caused by degenerative changes and is a major cause of chronic pain worldwide.

### Nociplastic pain

The Terminology Task Force of the International Association for the Study of Pain (IASP) recently proposed a new term, called nociplastic pain (18). Nociplastic pain describes a category for pain in which the mechanisms are not fully understood. However, there is an appreciation that there is increased CNS pain and sensory processing with altered pain modulation. It has been proposed that this form of pain is mechanistically and clinically distinct from nociceptive and neuropathic pain (19). Often, in these conditions there is significant pain reported by the sufferer but no noxious stimulus can be identified. In addition, there is minimal or no local tissue inflammation at the site of pain. Conditions which have features of nociplastic pain include fibromyalgia, temporomandibular pain and back pain.

### Pain sensitisation

Pain sensitisation is a pain characteristic that is often observed in different categories of pain (20) and is divided into central and peripheral features (21). Central sensitisation is a term that describes overlapping and related pain mechanisms which are due to altered sensory processing in the brain (22). Central sensitisation has been described as “an amplification of neural signalling within the central nervous system that elicits pain hypersensitivity” (23). Peripheral sensitisation is described as heightened pain sensitivity in a peripheral nerve outside the brain (24). Central sensitisation is also typified by continual activation of central brain pain networks and lack of response of endogenous analgesia (25). Central sensitisation is a major mechanism of nociplastic pain. However, nociplastic pain covers a wider range of features in addition to central sensitisation and is often diagnosed clinically in the apparent absence of actual or threatened tissue damage. Central sensitisation is also an important mechanism of neuropathic pain and can be observed in subacute pain.

### Pain in rheumatic and musculoskeletal diseases (RMD)

Pain perception is a complex measurable trait. A number of factors influence pain perception in RMD, including environmental exposures e.g., local injury, infection, smoking, increased body mass and multiple genetic factors (see discussion below). Several tools have been used in the clinic and in research studies to evaluate different forms of pain. These include a range of questionnaires to assess nociception and inflammatory pain. Self-reported pain intensity measures include the Visual Analogue Scale (VAS) (26) and the Numerical Rating Scale (NRS) (27). The impact of pain on physical function can be assessed using measures such as the Brief Pain Inventory BPI (28) and quality of life with measures such as Euroqol 5D (29). The impact of emotional factors e.g., anxiety and depression on pain can be measured using questionnaires such as the Hospital Anxiety and Depression Scale (HADS) (30). Neuropathic pain elements can be assessed using questionnaires (31) such as the Leeds Assessment of Neuropathic Symptoms and Signs (LANSS), Douleur Neuropathique en 4 questions (DN4), ID pain, Neuropathic Pain Scale (NPS), Brief Pain Inventory (BPI), painDETECT (32) and the Neuropathic Pain Questionnaire (NPQ) (33). In clinical practice, it would be more desirable to use questionnaires with a higher sensitivity and specificity (31). More recently, there has been interest in assessing nociplastic pain. Tools such as quantitative sensory testing (QST) have been used for evaluating central and peripheral pain sensitisation and are becoming more widely used but may be challenging clinically due to higher costs than using questionnaires (34). Neuroimaging tools e.g., functional magnetic resonance imaging (fMRI) (35) and spectroscopy (36) has also been deployed to evaluate brain regions involved in pain processing and to measure potential impacts of interventions e.g., drugs or behavioural changes (37). For back pain, the STarT Back screening tool has been validated to evaluate and stratify the likelihood of developing chronic pain after an acute episode and is used in clinical practice (38). The Oswestry Disability Index (39) and Roland Morris Disability Questionnaire (40) are also widely used to assess disability associated with back pain.

### Pain mechanisms in distinct rheumatic and musculoskeletal diseases (RMD)

In the next section, we discuss how a range of assessment tools are being used to stratify pain in RMD.

### Pain in inflammatory arthritis

The autoimmune inflammatory arthritides include disorders such as rheumatoid arthritis (RA), psoriatic arthritis (PsA) and axial spondyloarthritis (AxSpa). RA is common, affecting up to 1% of adult populations worldwide. Traditionally, pain mechanisms in RA, PsA and AxSpa have been shown to be mediated by nociceptive and inflammatory pathways (41). The local release of cytokines peripherally in the joint e.g., tumour necrosis factor alpha (TNF-α), interleukin-6 and other factors such as prostaglandins, leukotrienes and neuroptrophins such as Nerve Growth Factor (NGF), Substance P, engage with peripheral nociceptors in the joint and induce pain (42). Treatment of inflammatory arthritides has been transformed over the last few decades, with the introduction and use of both synthetic and biologic disease modifying anti-rheumatic drugs (DMARDs) which are targeted at suppression of pro-inflammatory pathways and activated cytokines (43). DMARD treatments for RA include targeted synthetic DMARDs e.g., methotrexate, sulfasalazine, hydroxychloroquine and in people where synthetic DMARDs are insufficient to control disease activity, biologic agents such as TNF-α inhibitors, rituximab, IL-6 receptor antagonists and Janus Kinase (JAK) Inhibitors may be prescribed. Although remission and treatment targets are met by many more patients than previously, there remains a high burden of disease in many patients, who have ongoing pain. People with inflammatory arthritides may well respond to analgesics e.g., NSAIDs which are prescribed to treat inflammatory pain (44). However, NSAIDs are usually prescribed at the lowest effective dose and for as short a duration as possible to minimise longer term side effects such as cardiovascular and renal morbidity (44). Despite control of inflammation, people with inflammatory arthritis may experience ongoing pain due to multiple factors, including pain sensitisation. Features of pain sensitisation can be evaluated using questionnaires, QST and neuroimaging (45, 46). A major question regarding pain sensitisation is whether neural networks can be “switched off” at some stage during treatment e.g., with early and tight control of inflammation, or whether development of pain sensitisation is an inevitable effect of the disease burden in certain individuals e.g., with risk factors such as anxiety, depression. It has been argued that there is some neuroplasticity in people with inflammatory conditions, which can be addressed using behavioural changes (47), pain management programmes (PMP) (37), antidepressants (48) or centrally-acting analgesics (49). Earlier recognition of pain sensitisation e.g., using QST, pain sensitisation questionnaires including LANSS, DN4, NPS, NPQ and painDETECT may be useful to recognise nociplastic pain and pain sensitisation early and consider methods to address pain management.

### Pain in osteoarthritis

Osteoarthritis (OA) is the most common arthritis worldwide, with a prevalence of over 10 million in the UK alone (50). Pain is one of the most common symptoms of OA. Recent studies have interrogated the mechanisms of pain in OA (51, 52). Traditionally, OA has been described as a condition affecting cartilage, with denudation of the cartilage surface as the condition progresses, eventually leading to underlying bone changes including osteophytes, bone sclerosis and joint space narrowing (53). Since cartilage is avascular and aneural, other joint structures must be responsible for causing pain. Major structural components of the joint demonstrated as mediators of pain include synovium and underlying bone—in particular, bone marrow lesions (BMLs). How do these findings relate to clinical assessment of pain in OA? In recent years it has been recognised that, in addition to inflammatory pain, sensitisation is an important feature of pain in OA and is an important factor for pain chronicity (54). Recent work has suggested that addressing both inflammatory and nociplastic pain components in OA e.g., inflammatory pain (due to synovitis) with non-steroidal anti-inflammatory drugs (NSAIDs) and therapies to address nociplastic pain e.g., pain management programmes, exercise and physiotherapy and/or centrally acting analgesics are critical to optimal pain management in OA (55, 56).

There is currently a huge unmet need to address pain management in OA. Potential novel therapeutic targets have been identified e.g., monoclonal antibodies targeted against Nerve Growth Factor (NGF) (57, 58). However, in conditions such as OA, nociceptive pain may be protective. The trials of anti-NGF monoclonal antibodies demonstrated that there was a phenomenon of rapidly progressive OA identified in a subgroup of participants who were also taking NSAIDs (59). Results from this trial show that complete blockage of nociceptive pain in some people, which led to complete pain relief in the trial, can also accelerate joint destruction (60). Therefore, addressing pain management with disease-modifying interventions such as anti-NGF monoclonal biologic therapies can also be problematic in some settings.

### Back pain

Back pain is a complex condition with several clinical features including pain, stiffness and reduced range of movement. In RMD, back pain is mostly non-specific and mechanical in nature. For most people who experience back pain worldwide, a specific nociceptive cause is not identified (61). When assessing a patient, it is important to exclude specific causes of back pain e.g., cancer, infection, trauma or inflammatory disease such as spondyloarthritis. If non-specific back pain is confirmed, then guidelines recommend a multi-disciplinary approach, including weight loss, exercise, psychological treatments and regular monitoring of symptoms (62, 63). A large part of long-term care in back pain involves self-management, and several strategies to ensure good uptake of this are important e.g., adherence to a regular exercise programme (64, 65).

Specific causes of back pain are rarer and can include a wide spectrum of conditions that cause back pain, including inflammatory causes such as inflammatory axial spondyloarthritis (AxSpa), traumatic injury, infection, cancer and spinal degenerative disease (62). People with fibromyalgia may also experience chronic back pain as a prominent feature of their condition. Where an inflammatory cause is the underlying problem e.g in AxSpa, a complete assessment for inflammatory, neuropathic or nociceptive pain can be performed. Various treatment options can be considered for inflammatory back pain, including NSAIDs, and in cases of AxSpa where NSAIDs have been insufficient to control pain, a range of biologic therapies can be considered, including TNF-α inhibitors and JAK inhibitors (62).

### Pain in fibromyalgia

Fibromyalgia has a high prevalence of 2%–4% in the general population, with a higher incidence in females (66). Fibromyalgia (FM) covers a range of symptoms, including widespread pain, sleep disturbance, poor concentration and memory. Studies of pain characteristics in FM have used a variety of tools to assess pain, including neuropathic pain questionnaires e.g., painDETECT and the neuropathic pain scale (67). Brain neuroimaging methods including functional brain neuroimaging have also demonstrated activation of central pain networks (66). There are genetic polymorphisms that have been reported more widely in FM, including the serotonin 5-HT_2A_ receptor, serotonin transporter, dopamine 4 receptor and *COMT* polymorphisms (68–70). Currently it is not understood how specific genetic polymorphisms might be associated with specific pain endotypes and their response to potential therapeutic interventions is not understood. Considering the high burden of disease chronic pain places on populations worldwide, further work is urgently needed to identify the relation between genetic changes, their clinical endotypes and identification of selective therapeutics based on endotypes.

### Why does pain persist, even when people are on treatment?

In RMD, patients may be in remission through suppression of inflammation, but chronic pain burden is high. Approaches to pain management are not optimised, may be considered late in an established diagnosis and access to care e.g., pain management programmes, is not universal.

The reasons for pain chronicity in RMD are multifactorial (71, 72). Chronic pain conditions can be associated with physical and psychological triggers. Both types of triggers can amplify pain and lead to ongoing psychological distress. Ongoing pain is a recognised issue observed in people treated for autoimmune conditions such as rheumatoid arthritis (71). Historically, physicians treating RMD may see evidence of good control of inflammatory markers such as Erythrocyte Sedimentation Rate (ESR) or C reactive protein (CRP). Suppression of inflammation in RMD assessed by ESR and CRP after treatment with disease-modifying anti-rheumatic drugs (DMARDs), including synthetic agents such as methotrexate and biologic agents including TNF-α modulators, IL-6 receptor antagonist and JAK-STAT pathway inhibitors e.g., baricitinib and tofacitinib (71) is a major objective of RMD treatments. In many autoimmune conditions such as rheumatoid arthritis, patients are provided with a management plan which aims to “treat-to-target”. For example, treatment to target in RA includes assessing tender and swollen joints, patient assessment of their disease control and inflammatory markers (ESR, CRP) and setting a treatment plan to control disease activity to a level using the parameters described for the Disease Activity Score (DAS28). Many patients treated with DMARDs nowadays achieve low levels of disease activity with low inflammatory markers (ESR, CRP), but may still report significant levels of pain (72). When inflammation is controlled but patients have ongoing pain, then fibromyalgia has been proposed as a potential mechanism for ongoing pain (14). Factors including ongoing activation of specific pro-inflammatory cytokines, depression and sleep disturbance can contribute to fibromyalgia in RA (14). Certain people may develop features of fibromyalgia early in the disease when chronic pain activation pathways may develop (73). There is increasing acceptance that fibromyalgia should be recognised and treated early in the context of existing RMD (74), which has recently been aided by a change in the classification criteria from a previous diagnosis of exclusion (75) to one that can exist in the presence of other painful conditions (76). Increased awareness and early monitoring by clinicians for ongoing pain is imperative so that patients can be offered early interventions (77).

There are some genetic polymorphisms which are linked with musculoskeletal pain, including several involved in serotonergic and adrenergic pathways (68–70). What is currently lacking is translation from genetic associations to the development of novel therapeutics and methods to assess pain, which could lead to more personalised treatment approaches for chronic pain in the future. Since there is now expanding evidence for the links between genetic risk factors for pain and specific pain disorders (see Table 1), researchers can aim to interrogate pathways which may have mechanisms with druggable therapeutic targets e.g., targeting COMT activity and/or elevated catecholamine levels can be treated with pharmacological agents that block both β2- and β3-adrenergic receptors (70). Although advances have been made in our understanding of the genetic risk factors and correlates for pain, further work is required to understand the clinical significance and therapeutic implications of research findings.

**Table 1:** Genes associated with rheumatic and musculoskeletal diseases.

Mapped genes/loci region	Gene functions	SNP	Outcome	PMID
*DCC*	Nociceptive pathways	rs4384683	Chronic back pain	30261039
*DCC*	Nociceptive pathways	rs62098013	Multisite chronic pain	31194737
*DCC*	Nociceptive pathways	rs72922230	Chronic back pain	33021770
*DCC*	Nociceptive pathways	18:50442591_TTTC_T	Multisite chronic pain	33830993
*FALEC; ADAMTSL4*	Nervous system development (*ADAMTSL4*)	rs59898460	Multisite chronic pain	31194737
*FALEC; ADAMTSL4*	Nervous system development (*ADAMTSL4*)	rs367563576	Chronic back pain	33021770
*FALEC; ADAMTSL4*	Nervous system development (*ADAMTSL4*)	rs59898460	Multisite chronic pain	33830993
*CA10; LINC01982*	Brain development (*CA10*)	rs12453010	Shoulder and neck pain	32246137
*CA10; LINC01982*	Brain development (*CA10*)	rs11079993	Multisite chronic pain	33830993
*CA10; LINC019842*	Brain development (*CA10*)	rs11079993	Multisite chronic pain	31194737
*EXD3*	Cell-cycle progression	rs73581580	Multisite chronic pain	31194737
*EXD3*	Cell-cycle progression	rs73581580	Genetic components of chronic musculoskeletal pain	32587327
*EXD3*	Cell-cycle progression	rs73581580	Multisite chronic pain	33830993
*FOXP2*	Brain development	rs12537376	Multisite chronic pain	31194737
*FOXP2*	Brain development	rs2049604	Shoulder and neck pain	32246137
*FOXP2*	Brain development	rs12705966	Genetic components of chronic musculoskeletal pain	32587327
*SLC39A8*	Inflammation	rs13135092	Multisite chronic pain	31194737
*SLC39A8*	Inflammation	rs13107325	Genetic components of chronic musculoskeletal pain	32587327
*SLC39A8*	Inflammation	rs13135092	Multisite chronic pain	33830993
*SOX5*	Chondrogenesis	rs12310519	Chronic back pain	30261039
*SOX5*	Chondrogenesis	rs12310519	Chronic back pain	30747904
*SOX5*	Chondrogenesis	rs12308843	Chronic back pain	33021770
*LINC01065; LINC00558*	Not known	rs1443914	Multisite chronic pain	31194737
*LINC01065; LINC00558*	Not known	rs34003284	Multisite chronic pain	33830993
*RNF123; AMIGO3; GMPPB*	Brain development, synapse assembly (*AMIGO3*), cell cycle progression (*RNF123*)	rs7628207	Genetic components of chronic musculoskeletal pain	32587327
*RNF123; AMIGO3; GMPPB*	Brain development, synapse assembly (*AMIGO3*), cell cycle progression (*RNF123*)	rs1491985	Chronic widespread pain	33926923
*RNF123; AMIGO3; GMPPB*	Brain development, synapse assembly (*AMIGO3*), cell cycle progression (*RNF123*)	rs7628207	Multisite chronic pain	31194737
*C6orf106*	Inflammation	rs6907508	Multisite chronic pain	31194737
*C6orf106*	Inflammation	rs151060048	Multisite chronic pain	33830993
*C8orf34*	Not known	rs1865442	Chronic back pain	30747904
*C8orf34*	Not known	rs7834973	Chronic back pain	33021770
*CCDC26; GSDMC*	Lumbar disc degeneration	rs7814941	Chronic back pain	30747904
*CCDC26; GSDMC*	Lumbar disc degeneration	rs7833174	Chronic back pain	30261039
*FAF1*	Apoptosis	rs10888692	Multisite chronic pain	31194737
*FAF1*	Apoptosis	rs35072907	Multisite chronic pain	33830993
*GDF5*	Osteoarthritis	rs143384	Chronic knee pain	31482140
*GDF5*	Osteoarthritis	rs143384	Genetic components of chronic musculoskeletal pain	32587327
*SLC24A3*	Electrical conduction	rs2424248	Multisite chronic pain	31194737
*SLC24A3*	Electrical conduction	20:19709268_AAAAT_A	Multisite chronic pain	33830993
*SOX6*	Chondrogenesis	rs61883178	Multisite chronic pain	31194737
*SPOCK2*	Neurogenesis	rs1678626	Chronic back pain	33021770
*SPOCK2*	Neurogenesis	rs3180	Chronic back pain	30747904
*FCGBP*	Maintenance of the mucosal structure	rs17796312	Chronic widespread pain	22956598
*SP4*	DNA-binding transcription factor activity	rs73271865	Temporomandibular disorder	28081371
*SP4*	DNA-binding transcription factor activity	rs7798894	Multisite chronic pain	31194737

Table adapted from Li et al., (78).

### Genetic basis of pain

Many RMD have genetic risks with common and rare genetic variants which can contribute to specific endotypes. However, the causes of pain may differ between and within RMDs. Gaining a better understanding of how genetics influence musculoskeletal pain may yield new therapeutic avenues through prediction, prevention, and personalization. Producing reliable genetic association results that give meaningful mechanistic insights depends on appropriate specification of a trait or phenotype of interest (79). In common with other complex traits such as psychiatric disorders, genome-wide association studies (GWAS) that seek to understand the genetic determinants of pain can be hampered by aetiological and phenotypic heterogeneity (80). One approach to this is to group individuals into pain phenotypes based on a presumed shared disease mechanism. As described above, an example of mechanistic grouping of pain is into nociceptive, neuropathic, and nociplastic pain, though in practice these commonly overlap (81, 82). Accurate identification of these pain mechanisms also requires deep pain phenotyping, which is feasible in smaller scale genetic association studies (83), but is often not available in large-scale datasets required for conducting sensitive GWAS for complex polygenic diseases. A partial exception is neuropathic pain identified using questionnaires such as the Douleur Neuropathique 4 (84), though this does not characterise pain in as much depth as endotyping based on, for example, quantitative sensory testing (QST). In practice, the pain phenotype groupings used for genetics research are commonly based on anatomical site, duration, or primary pain syndrome. These may be more reliably captured using routinely collected health data or self-report; examples include chronic back pain (85), multi-site or widespread chronic pain (86–88), fibromyalgia (89), and sciatica (90). A potential limitation of site-based phenotypes is that they may be biased towards identifying variants associated with a primary pain-causing pathology (e.g., osteoarthritis) rather than pain itself (91).

Despite multifarious methodological challenges, a large number of variants have been identified which may be implicated in the development of musculoskeletal pain (Table 1). The weight of evidence to date would seem to suggest that genetics influence pain predominantly through the CNS (92–94). This may be particularly the case for chronic pain, with some recent work suggesting genetic variants associated with chronic, but not acute, back pain are brain-specific (93). The same study also found that acute back pain is less heritable than chronic pain. Similarly, pain sensitisation driven by active inflammation at a knee affected by OA may have different genetic drivers to distal sensitisation (83). These findings together can be seen to corroborate the idea that acute pain is almost exclusively nociceptive (95), secondary to actual or potential tissue damage caused by environmental factors rather than under direct genetic influence. However, the way acute pain is experienced may be modulated by pain sensitivity, the genetic underpinnings of which have been studied using subjective and objective measures of sensitivity and susceptibility (94). CNS-specific variants that have been implicated in pain include those that affect serotonergic, adrenergic, and glutamatergic pathways (96, 97).

Some more recent work uses a novel approach to move beyond grouping individuals based on a site-based phenotype (80). The approach uses principal component analysis to abstract a series of “genetically independent phenotypes” from GWAS summary statistics from different site-based chronic musculoskeletal pain phenotypes. This allows pooling of individuals reporting pain at different sites, and ostensibly observation of shared genetic determinants for the general tendency to chronic musculoskeletal pain. This work identified several variants in individuals of white European ancestry that are also reported by GWAS of chronic multisite pain (88), and further validated several of these variants in cohorts of other ethnicities. Of note, in common with other site-specific musculoskeletal pain studies, this still identifies loci that have known associations with osteoarthritis. Different approaches for looking into shared genetic associations across different pain phenotypes are also under exploration (98). While not without limitations, approaches such as these may complement research which uses deep, mechanism-based pain phenotyping within smaller cohorts, and large-scale studies using phenotypes identified with more typical methods.

### Pharmacogenomics in pain management

There are several pharmacogenetic variants which may influence response to—or harm caused by—certain analgesic medicines. These include metabolic enzymes (e.g., CYP2D6, COMT), efflux pumps (e.g., ABCB1), and receptors (e.g., OPRM1) (99). While CYP2D6 is already considered to have actionable variants with implications for the selection of opioid type and dose, with further advancements and reduced costs of pharmacogenetic testing, more genes and variants may prove actionable in the future, providing further guidance for personalizing and optimizing medical pain management (100).

### Pharmacological approaches to management

**Figure 3:**
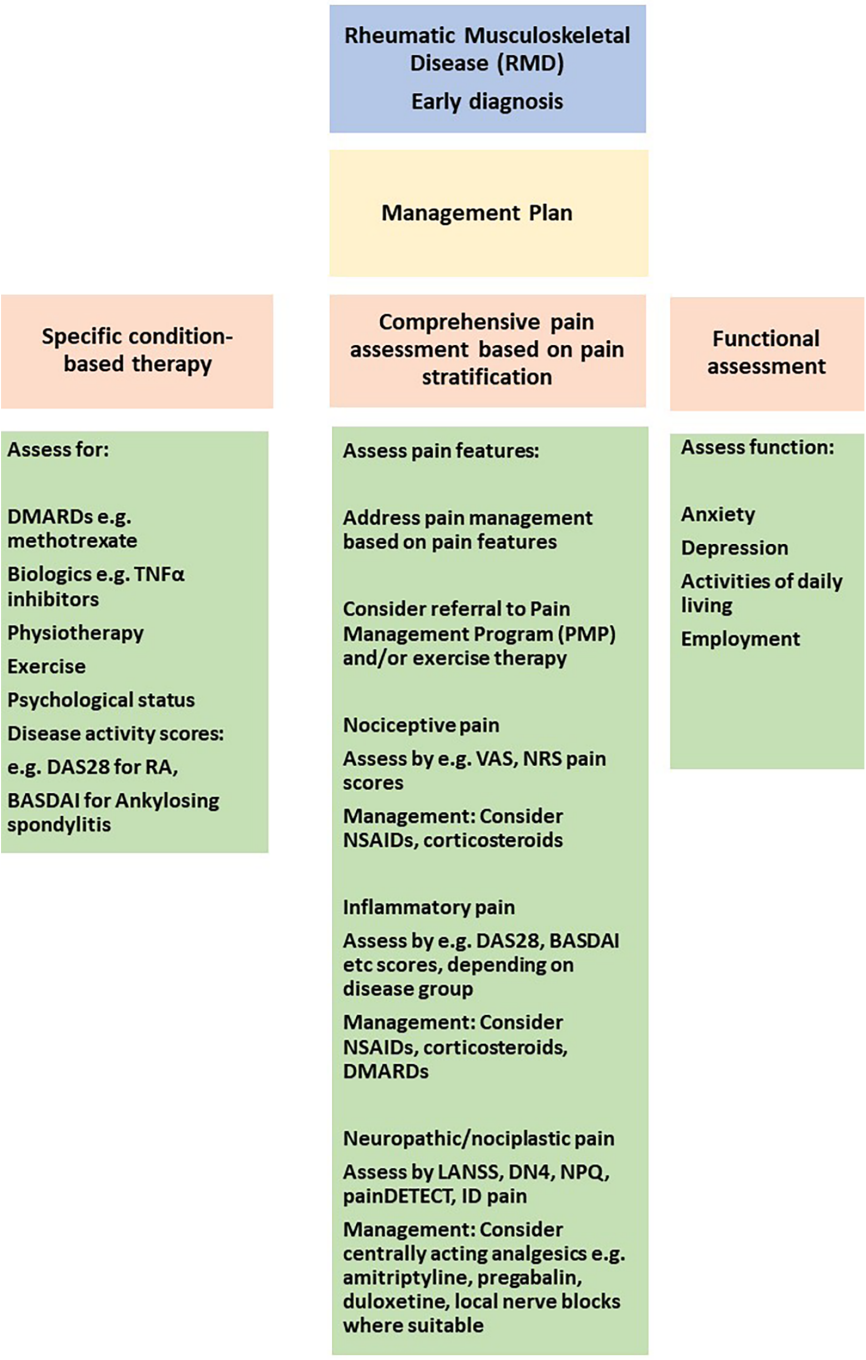
Approaches to pain stratification and management in the clinic. VAS, visual analogue scale; NRS, numerical rating scale; DAS28, disease activity score 28 for rheumatoid arthritis; BASDAI, bath ankylosing spondylitis disease activity index; LANNS, leeds assessment of neuropathic symptoms and signs, DN4, douleur neuropathique; NPQ, neuropathic pain questionnaire; TNF, tumor necrosis factor alpha.

A pharmacological approach to chronic pain management can be formulated after a full clinical assessment based on patient needs. Assessing the individual patient's needs with a full history and clinical assessment, aided by relevant questionnaires, can be used to evaluate if pharmacological intervention is warranted based on a holistic assessment (Figure 3).

For fibromyalgia and CWP, current NICE (National Institute for Health and Care Excellence) guidelines recommend that a multidisciplinary approach is desirable, including advice about exercise, talking therapies with pharmacological approaches to nociplastic pain management including amitriptyline, citalopram, duloxetine, fluoxetine, paroxetine or sertraline (101). Although NICE guidelines do not recommend opioids, NSAIDs, paracetamol or gabapentinoids for chronic primary pain (101), EULAR (European Alliance of Associations for Rheumatology) guidelines have included tramadol and the antiepileptic pregabalin, although the level of evidence for efficacy was low (102).

For the management of lower back pain, current NICE guidelines (63) recommend a multi-disciplinary approach that includes exercise, weight loss and advice about self-management, including diet, activity and symptom monitoring. Psychological support can also be beneficial such as cognitive behavioural therapy (CBT) and pain management programmes (PMP). In specific cases, NSAIDs and weak opioids may be of benefit. NICE guidelines do not recommend gabapentinoids, anti-epileptics, corticosteroids or benzodiazepines for back pain (63).

For OA, NICE guidelines (103) and EULAR guidelines (104) include NSAIDs (topical followed by oral if necessary) and potentially corticosteroid injections for pain management of nociceptive and nociplastic pain components. Typical doses of NSAIDs include ibuprofen up to 1,200 mg daily in divided doses or naproxen 1,000 mg daily in divided doses (103). NSAIDs should be taken at the lowest dose which is efficacious for the patient for as short a duration as possible e.g., due to flare-ups of symptoms and a proton pump inhibitor can also be prescribed concomitantly with NSAIDs to minimise gastrointestinal side-effects. Opioids are not recommended in OA due to low evidence.

In autoimmune-mediated inflammatory disorders including RA and axial spondyloarthritis, NICE guidelines recommend NSAIDs may be used for pain management, particularly when DMARDs are insufficient to control symptoms (105, 106). If people with inflammatory RMD have clinical and biochemical control of inflammation, but still have ongoing pain symptoms consistent with fibromyalgia or CWP, then it is suggested that pain management can follow recommended guidelines for fibromyalgia and CWP (71, 102, 103).

Regarding future treatments currently in trials, cannabinoids in fibromyalgia and other RMD are being considered, several of which are currently in progress (107, 108). While there are dozens of phytocannabinoids which may have future therapeutic value, recent clinical trial evidence would seem to suggest that cannabidiol, the most studied non-psychoactive cannabinoid, does not confer additional pain relief in knee OA (109). Drugs targeted at inhibiting inflammatory pathways in OA are also being conducted, including pentosan polysufate, targeting the NFKB pathway (110). A novel combination therapy trial is currently underway using a combination of LNA043, which targets cartilage damage and canakinumab, which targets inflammation using the IL-1-receptor antagonist canakinumab in OA (111).

## Discussion

In this review we have summarised current understanding of pain mechanisms in RMD, approaches to achieve pain stratification by defining endotypes, genetic risk factors and response to specific pharmacological approaches. A deeper understanding of pain mechanisms and management could be used to consider pain management pathways in clinical practice. Front and foremost is to make a definitive diagnosis of the RMD. Following this, a firm diagnosis will trigger a discussion with the patient about therapeutic interventions, including pharmacological and non-pharmacological approaches. It is important at the first patient encounter to conduct a comprehensive clinical assessment, including a physical examination and pain evaluation as summarised in Figure 3. By combining clinical assessments with questionnaires assessing specific pain components i.e., nociceptive, inflammatory, neuropathic and nociplastic, the patient can be stratified for their pain perception. Figure 3 demonstrates how distinct questionnaires can be used to stratify for specific pain components.

In the initial stages of diagnosis, it is important to control inflammation e.g., with corticosteroids, DMARDs as appropriate. Assessment of response to DMARDs can be made by disease-specific scores e.g., DAS28, BASDAI (see Figure 3). Pain management is often with NSAIDs in the initial stages. Once inflammation is better controlled, the requirement for NSAIDs/corticosteroids should reduce as the effect of DMARD therapy sets in. More long-term for chronic pain management, potential pharmacological interventions can be considered depending on the mechanisms mediating pain e.g., NSAIDs for inflammatory pain, opioids for neuropathic pain or centrally-acting analgesics for nociplastic pain. Non-pharmacological interventions, in particular, early exercise and physiotherapy-based interventions can assist is maintaining function, muscle mass and reducing pain. The information collected should assist in developing a pain management plan which is personalised for each patient. It is only through individual patient pain assessments that a personalised and tailored approach can be applied for long-term management and achieving sustained disease control.
